# Haematopoietic and cardiac GPR55 synchronize post-myocardial infarction remodelling

**DOI:** 10.1038/s41598-021-93755-y

**Published:** 2021-07-13

**Authors:** Sarah-Lena Puhl, Michael Hilby, Michael Kohlhaas, Linus M. Keidel, Yvonne Jansen, Michael Hristov, Jakob Schindler, Christoph Maack, Sabine Steffens

**Affiliations:** 1 Institute for Cardiovascular Prevention (IPEK), Ludwig-Maximilians-Universität (LMU) Munich, Pettenkoferstr. 9, 80336 Munich, Germany; 2 German Centre for Cardiovascular Research (DZHK), Partner Site Munich Heart Alliance, Munich, Germany; 3 Comprehensive Heart Failure Center, University Clinic Würzburg, Würzburg, Germany; 4 Medical Clinic I, University Clinic Würzburg, Würzburg, Germany

**Keywords:** Immunology, Cardiology, Molecular medicine, Pathogenesis

## Abstract

While classical cannabinoid receptors are known to crucially impact on myocardial infarction (MI) repair, a function of the cannabinoid-sensitive receptor GPR55 herein is poorly understood. We investigated the role of GPR55 in cardiac physiology and post-MI inflammation and remodelling. Global GPR55−/− and wildtype (WT) mice were basally characterized or assigned to 1, 3 or 28 days permanent MI and subsequently analysed via pro-inflammatory and pro-hypertrophic parameters. GPR55−/− deficiency was basally associated with bradycardia, increased diastolic LV volume and sarcomere length and a subtle inflammatory phenotype. While infarct size and myeloid cell infiltration were unaffected by GPR55 depletion, acute cardiac chemokine production was prolonged post-MI. Concurrently, GPR55−/− hearts exhibited a premature expansion of pro-reparative and phagocytic macrophages paralleled by early up-regulation of extracellular matrix (ECM) factors 3 days post-MI, which could be mimicked by sole haematopoietic GPR55 depletion. Moreover, global GPR55 deficiency mitigated MI-induced foetal gene re-programming and cardiomyocyte hypertrophy, culminating in aggravated LV dilatation and infarct expansion. GPR55 regulates cardiac homeostasis and ischaemia responses by maintaining adequate LV filling and modulating three crucial processes post-MI: wound healing kinetics, cardiomyocyte hypertrophy and maladaptive remodelling.

## Introduction

Coronary artery disease and MI remain the leading causes of death worldwide. The extent of contractile myocardium loss and maladaptive LV remodelling, including infarct thinning and expansion combined with LV volume-overload and dilation, determine the remaining LV function and thereby the patient’s long-term prognosis post-MI. Early after onset of MI, ischaemic cardiomyocytes become necrotic in the infarct area, but also apoptotic in the infarct and border zones while surviving cardiomyocytes in the remote area initiate pro-hypertrophic alterations to provide hemodynamic compensation for the loss of contractile tissue^1,2^. Simultaneously, tissue-resident immune and non-immune cells produce pro-inflammatory factors such as CC-chemokine ligand 2 (CCL2), interleukins (IL) IL-1β and IL-6, tumor necrosis factor α (TNFα) and oxidative stress^3,4^. Neutrophils and circulating monocyte-derived macrophages are recruited in large numbers to the infarct area, where they phagocytose necrotic cardiomyocytes and release proteases, in particular matrix metalloproteinase 9 (MMP-9) which breaks down collagen and facilitates further infiltration of inflammatory cells^5–10^.

A simplified way to, at least, roughly characterize macrophages is to decipher between pro-inflammatory and alternatively activated, pro-reparative subsets. The latter, starting to accumulate in the infarcted heart at day 3 express F4/80, CD64, CD206, arginase 1 and myeloid-epithelial-reproductive tyrosine kinase (MERTK)^11–13^. Those macrophages secrete anti-inflammatory, pro-fibrotic and pro-angiogenic factors, such as IL-10, transforming growth factor β, vascular endothelial growth factor, and MMPs—thereby, resolving inflammation and promoting repair mechanisms^10,14,15^. The post-MI inflammatory resolution process must be timely and tightly regulated to pave the way for appropriate wound healing and formation of a stable scar^16^.

Classical cannabinoid receptors contribute to post-MI wound healing via their function on immune and cardiovascular cells, with CB1 activation shown to be rather pro-inflammatory and CB2 promoting anti-inflammatory effects^17–19^. GPR55 was originally reported as a putative novel receptor for endocannabinoids and cannabinoid-related ligands, although some controversy exists about its cannabinoid binding activities^20^. An additional endogenous ligand for this receptor is lysophosphatidylinositol (LPI)^20^. The role of GPR55 in wound healing following cardiac injury is poorly investigated. In metabolic diseases, tissue expression of GPR55 and circulating levels of LPI are elevated^21,22^. GPR55 expression has been reported on several immune cell subtypes. In human peripheral monocytes and natural killer cells, GPR55 signalling increases cytokine production, cell cytotoxicity, decreases monocyte endocytosis and synergizes with CB2 to promote neutrophil migration^23^. As to the heart, low-level *Gpr55* mRNA expression has been detected in the rodent myocardium and in isolated neonatal cardiomyocytes^24^, while lack of GPR55 has been reported to affect cardiac contractility by impairing β_1_-adrenoceptor responsiveness^25^. Moreover, a correlation between intracellular Ca^2+^ mobilization and GPR55 stimulation with cannabinoids or LPI was demonstrated in isolated neonatal cardiomyocytes^24^. In rat hearts ex vivo, LPI infused directly via the coronary circulation 10 min prior to ischaemia and subsequent reperfusion (I/R) increased infarct size^26^. In contrast, administration of cannabidiol (CBD), a GPR55 antagonist, has been shown to reduce infarct size post I/R in rats in vivo^27,28^. Another study reported an improved cerebral blood flow following experimental stroke when mice were injected with CBD^29^. The rising popularity of prescription-free CBD together with the clinical application of the US Food and Drug Administration-approved CBD drug Epidiolex emphasize the urge to elucidate how lack of functional GPR55 signalling affects the cardiovascular system.

In the present study we characterized the cardiac and immunological phenotype of naïve GPR55 deficient mice and investigated whether lack of GPR55 affects infarct size, wound healing kinetics and concurrently maladaptive remodelling post-MI. To elicit the effect of GPR55 on acute inflammation kinetics, we examined the immune profile of GPR55 deficient mice under naïve conditions and after 1 and 3 days of permanent MI. Moreover, we investigated the impact of GPR55 deficiency on early hypertrophy as well as on long-term structural and functional outcome 28 days post-MI. 

## Results

### GPR55 deficiency leads to bradycardia, LV dilatation, pre-stretch and promotes a pro-inflammatory phenotype

We first confirmed *Gpr55* mRNA expression in different organs of WT mice. Spleen and bone marrow showed the highest transcript levels, while low level receptor expression was noted in heart, liver, kidney and perigonadal visceral adipose tissue (see Supplementary Fig. S1). The analysis of sorted cardiac cells revealed detectable *Gpr55* expression in all subsets isolated, including resident cardiac macrophages, cardiomyocytes and endothelial cells with fibroblasts exhibiting the lowest expression level (see Supplementary Fig. S1).

**Figure 1:**
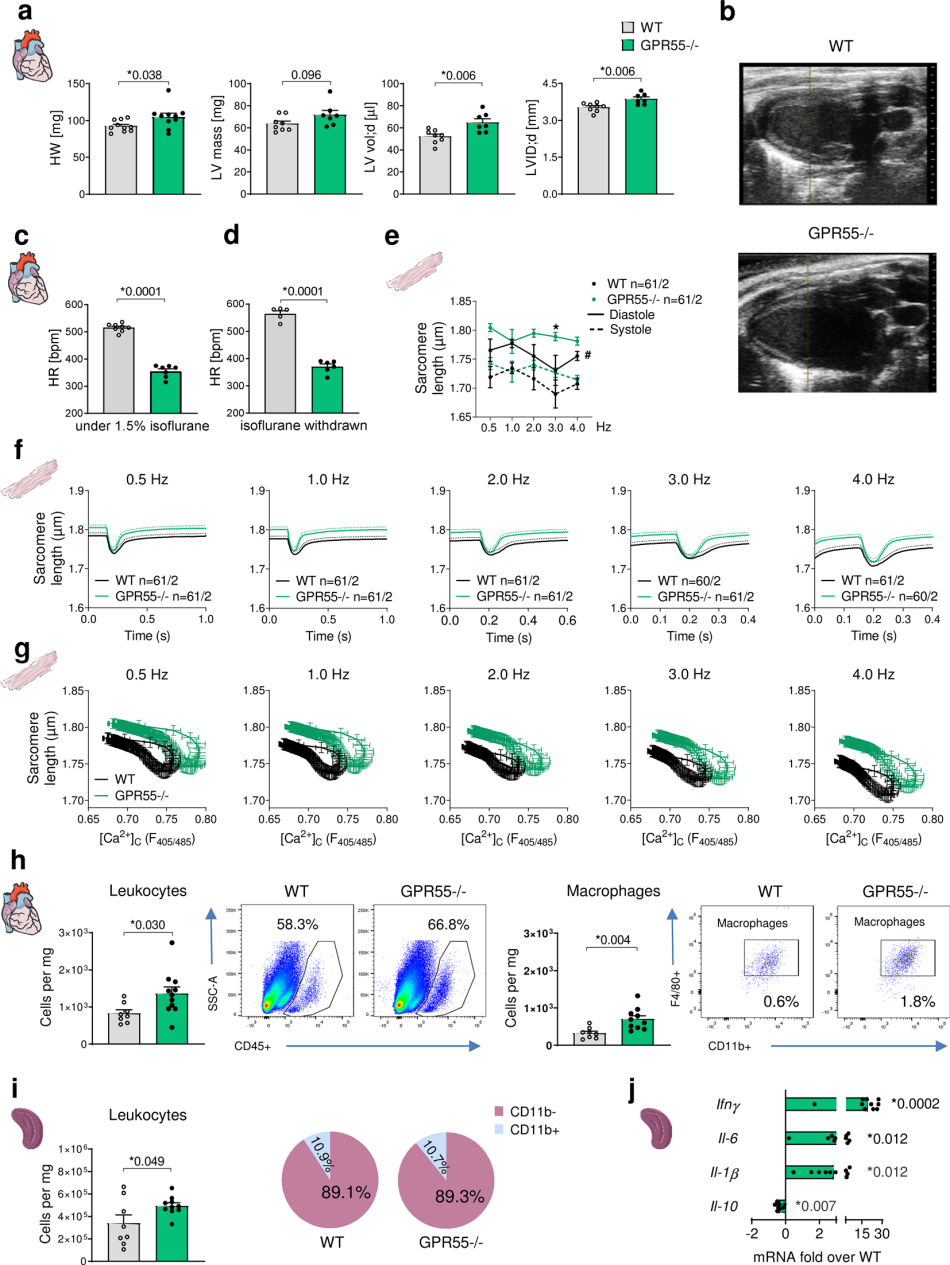
Baseline characterization of GPR55 deficient mice. (**a**) Basal cardiac phenotype of WT and GPR55−/− mice, aged 8 weeks, as determined by gravimetry and echocardiography (n = 8–11/group). (**b**) Representative echocardiographic B-Mode projections of WT and GPR55−/− hearts. Basal heart rate of WT and GPR55−/− mice (**c**) under 1.5% isoflurane inhalation (n = 7–8/group) and (**d**) under conscious conditions upon isoflurane withdrawal (n = 5–6/group). (**e**) Sarcomere length in isolated WT and GPR55−/− cardiomyocytes during diastole (solid lines) and systole (dotted lines) under increasing pacing frequencies. Circles indicate mean ± SE; ^#^p < 0.05, two-way ANOVA with Bonferroni’s post hoc test (*p < 0.05 vs. WT); n indicates number of isolated cardiomyocytes from 2 mice. (**f**) Sarcomere length recordings from isolated WT and GPR55−/− cardiomyocytes under increasing pacing frequencies over time. Solid lines indicate mean + SE (dotted line). (**g**) Ca^2+^ dependent sarcomere length in WT and GPR55−/− cardiomyocytes. (**h**) Basal LV leukocyte counts and herein macrophage numbers in WT and GPR55−/− mice detected via flow cytometry, and representative dot plots indicating respective leukocyte (CD45+) and macrophage (CD11b+ F4/80+) counts as percentage of single cells (n = 8–12/group). (**i**) Basal splenic leukocyte counts and relative distribution of myeloid (CD11b+) and lymphoid (CD11b-) cells in WT and GPR55−/− mice detected via flow cytometry (n = 8–12/group). (**j**) Basal splenic chemokine gene expression in GPR55−/− mice as fold over WT (n = 8–12/group). Bars and squares indicate mean ± SE (Comparison between two groups: Student’s t-test).

In naïve mice, GPR55 deficiency was associated with increased body weight, heart weight, LV mass and enlarged end-diastolic, but not end-systolic left ventricular volume and diameter (LVID) in the presence of a significantly decreased heart rate (Fig. 1a–c, Table 1) without changes in the functional parameters cardiac output, left ventricular (LV) ejection fraction (EF) and fractional shortening (FS) (see Supplementary Table S1). Of note, GPR55−/− mice were not only bradycardic under isoflurane inhalation, but also after isoflurane withdrawal when being awake (Fig. 1d). The increased diastolic volume in the absence of GPR55 was accompanied by enhanced atria and lung weight under baseline conditions per se, but not after normalisation against body weight (Table 1). Moreover, plasma albumin level and plasma volume were unaffected by the receptor deficiency (see Supplementary Fig. S2). The dilated phenotype in the absence of GPR55 therefore does most presumably not originate from excessive fluid accumulation, but rather from altered diastolic LV wall-stretch and filling dynamics. In support of this hypothesis, cardiomyocytes isolated from GPR55−/− mice exhibited an enhanced diastolic sarcomere length under physiological pacing frequencies (Fig. 1e–g) and a distinct reduction in time from peak contraction to 50% and 90% relaxation under each frequency applied when compared to WT cardiomyocytes (see Supplementary Fig. S3). On the contrary, while Ca^2+^ transient amplitude was enhanced in GPR55−/− myocytes, diastolic Ca^2+^ and time from peak tension to 50% and 80% Ca^2+^ decline were completely unaffected by GPR55 deficiency (see Supplementary Fig. S3). The Ca^2+^-contraction-relations clearly indicate that the observed contractile changes originate from a direct impact of GPR55 on myofilament calcium sensitivity. Herein, we confirm for the first time a cell-specific and contractile, myofilament function of GPR55 in adult murine cardiomyocytes.

**Table 1:** Basal phenotype of female mice aged 8 weeks, determined via gravimetry.

	WT	GPR55−/−
**Body dimensions**		
n	8	11
Body weight [g]	18.3 ± 0.2	21.7 ± 0.8*
**Heart chamber weights**		
n	8	11
Heart [mg]	90.9 ± 2.4	101.9 ± 2.6*
Atria [mg]	6.9 ± 0.4	8.6 ± 0.5*
**Wet organ weights**		
n	8	11
Lungs [mg]	120.0 ± 7.6	153.8 ± 9.5*
Liver [mg]	1001 ± 28	933 ± 45
Kidney [mg]	94.5 ± 3.2	114.2 ± 3.6*
Spleen [mg]	68.0 ± 2.1	70.2 ± 5.4
**Dry organ weights**		
n	8	11
Lungs [mg]	27.3 ± 0.7	31.5 ± 1.0*
Liver [mg]	329 ± 11	314 ± 14
Kidney [mg]	26.0 ± 0.5	28.5 ± 1.7
**Wet organ weights normalized to body weight [mg/g]**		
n	8	11
Heart	5.0 ± 0.1	4.7 ± 0.2
Lungs	6.6 ± 0.4	7.3 ± 0.7
Spleen	3.7 ± 0.1	3.2 ± 0.2*

Mean ± SE, *p < 0.05 vs. WT, student’s t-test.

To determine a potential impact of GPR55 on immune homeostasis, basal cardiac and splenic immune cell composition was assessed by flow cytometry. GPR55 deficient hearts exhibited a significantly higher abundance of leukocytes in general and, in particular, of macrophages and neutrophils compared to WT hearts (Fig. 1h, see Supplementary Fig. S2). This was accompanied by distinct up-regulation of *Mmp-9*, known to be expressed by macrophages and neutrophils, and the chemokine *Ccl2* (see Supplementary Fig. S2). Moreover, GPR55−/− mice had increased leukocyte numbers in lymphoid organs, while ratios between splenic myeloid (CD11b+) and lymphoid (CD11b−) cells were comparable between the genotypes (Fig. 1i). The increased leukocyte (CD45+) abundance in spleens of GPR55−/− mice was associated with elevated transcript levels of the pro-inflammatory cytokines *Il-1β*, *Il-6* and *Ifnγ* and a concurrent reduction of the anti-inflammatory marker *Il-10* (Fig. 1j). We may speculate that the observed impact on pro-inflammatory features and processes partially result from a volume-induced stretch of the myocardium in the absence of GPR55. However, a contribution of GPR55 to the development of LV volume overload and the associated immune response requires further investigation of the receptor in experimental overload settings to underpin our assumptions.

### GPR55 deficiency does not affect initial infarct size, neutrophil or monocyte infiltration kinetics post-MI

**Figure 2:**
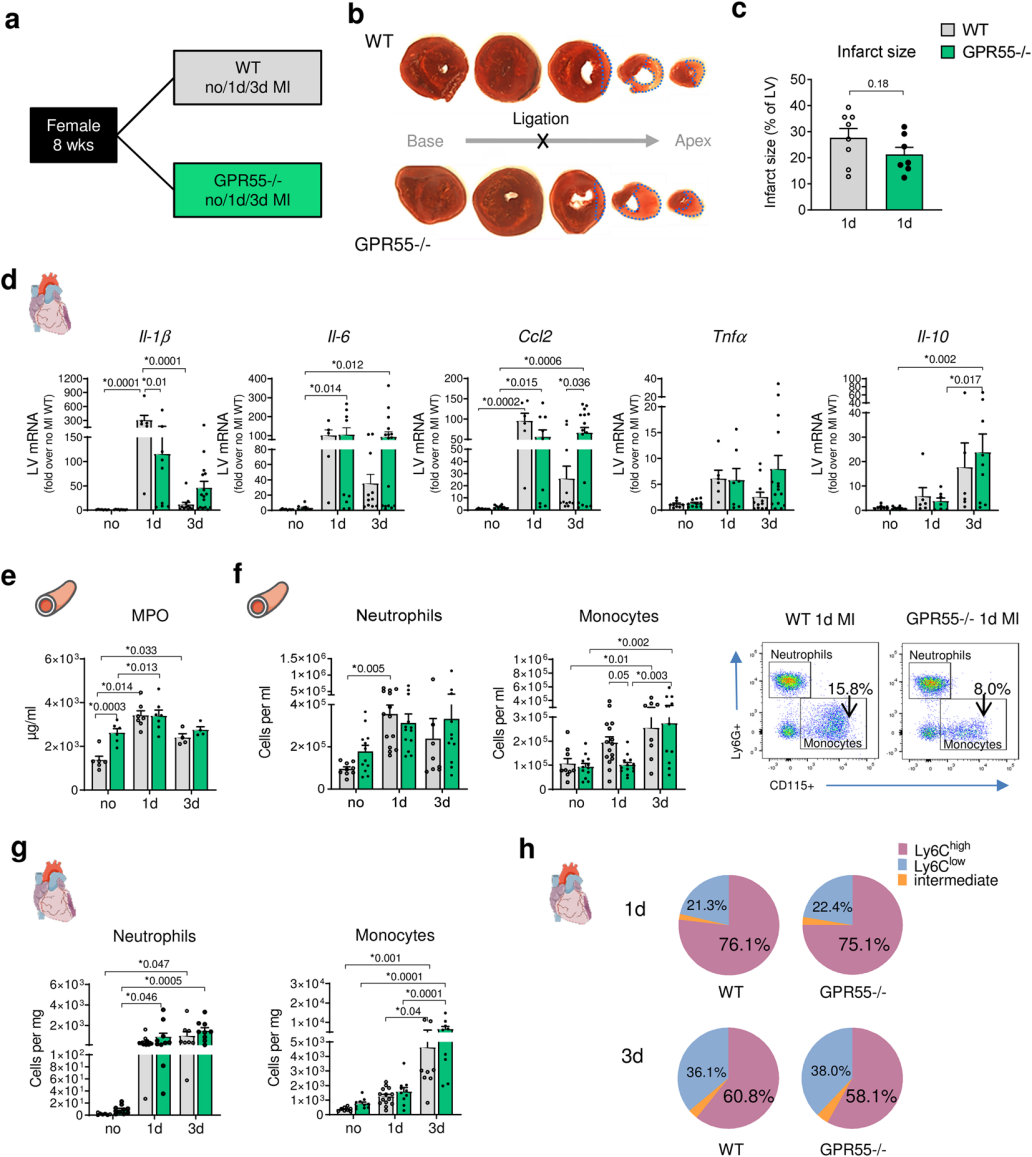
Impact of GPR55 deficiency on acute inflammatory response following MI. (**a**) Study design to explore the effect of GPR55 deficiency on acute post-MI wound healing. WT and GPR55−/− mice were randomly assigned to baseline characterization (no MI) or to MI via permanent LAD ligation for 1 or 3 days (d). (**b**) Representative TTC stained consecutive heart slices from base to apex with dotted blue lines indicating the infarct area measured and (**c)** quantification of infarct sizes 1 day post-MI in WT and GPR55−/− hearts (student’s t-test). (**d**) LV gene expression in WT and GPR55−/− mice relative to baseline WT. (**e**) Plasma MPO levels in WT and GPR55−/− mice, assessed via ELISA. (**f**) Total counts of circulating neutrophils and monocytes in WT and GPR55−/− mice, quantified via flow cytometry, and representative dot plots indicating respective monocyte counts (CD115+ Ly6G−) as percentage of single cells. (**g**) Neutrophil and monocyte counts in WT and GPR55−/− hearts. (**h**) Relative distribution of Ly6C^high, low^ and intermediate monocytes post-MI in WT and GPR55−/− mice. Bars indicate mean ± SE (n = 5–11/group) (Two-way ANOVA with Sidak’s post hoc test).

To investigate the role of GPR55 during acute inflammation and subsequent wound healing following MI, global GPR55−/− mice and their WT littermates were assigned to left anterior descending artery (LAD) ligation to induce a permanent MI for 1 or 3 days (Fig. 2a). Naïve, age-matched WT and GPR55−/− littermates served for baseline comparison (no MI). First, we confirmed comparable initial infarct sizes at day 1 post-MI between the genotypes, as assessed via TTC staining of the heart (Fig. 2b,c).

As expected, 1 day after MI onset WT mice exhibited a pronounced acute inflammatory response at the site of injury in the LV, as shown by distinct up-regulation of *Il-1b*, *Ccl2*, *Il-6* and *Tnfα* transcription compared to their corresponding no MI group. This acute response was partly mitigated in GPR55−/− mice at this time point (Fig. 2d). While the initial burst in cardiac cytokine and chemokine expression in WT decreased 3 days post-MI, these markers remained elevated in GPR55 deficient mice. Moreover, lack of GPR55 significantly up-regulated the anti-inflammatory cytokine *Il-10* at this time-point.

Next, we assessed the impact of GPR55 deficiency on cardiac and circulating myeloid counts as a measure of their release and recruitment to the infarcted heart. WT mice showed the expected MI triggered increase in circulating neutrophil counts and plasma MPO levels. In contrast, the sustained subtle volume-overload in GPR55−/− mice under no MI conditions was already associated with blood neutrophil counts and activity comparable to the post-MI WT phenotype. This could not be further potentiated by the acute ischaemic injury (Fig. 2e, f, see Supplementary Fig. S2). In addition, LV infiltrating neutrophils during the acute innate immune response, which peaked 1 to 3 days post-MI, remained unaffected by GPR55 deficiency (Fig. 2g). Blood monocyte numbers in GPR55−/− mice were slightly lower at day 1, yet comparable 3 days post-MI. Moreover, neither cardiac monocyte counts in general, nor ratio between classical Ly6C^high^ and non-classical Ly6C^low^ subsets or splenic mobilization thereof was affected by the absence of GPR55 during acute inflammation (Fig. 2f-h, see Supplementary Fig. S4). These observations suggest GPR55 not to impact on myeloid cell recruitment to the infarcted myocardium.

### GPR55 deficiency promotes cardiac expansion of pro-repair macrophages post-MI

**Figure 3:**
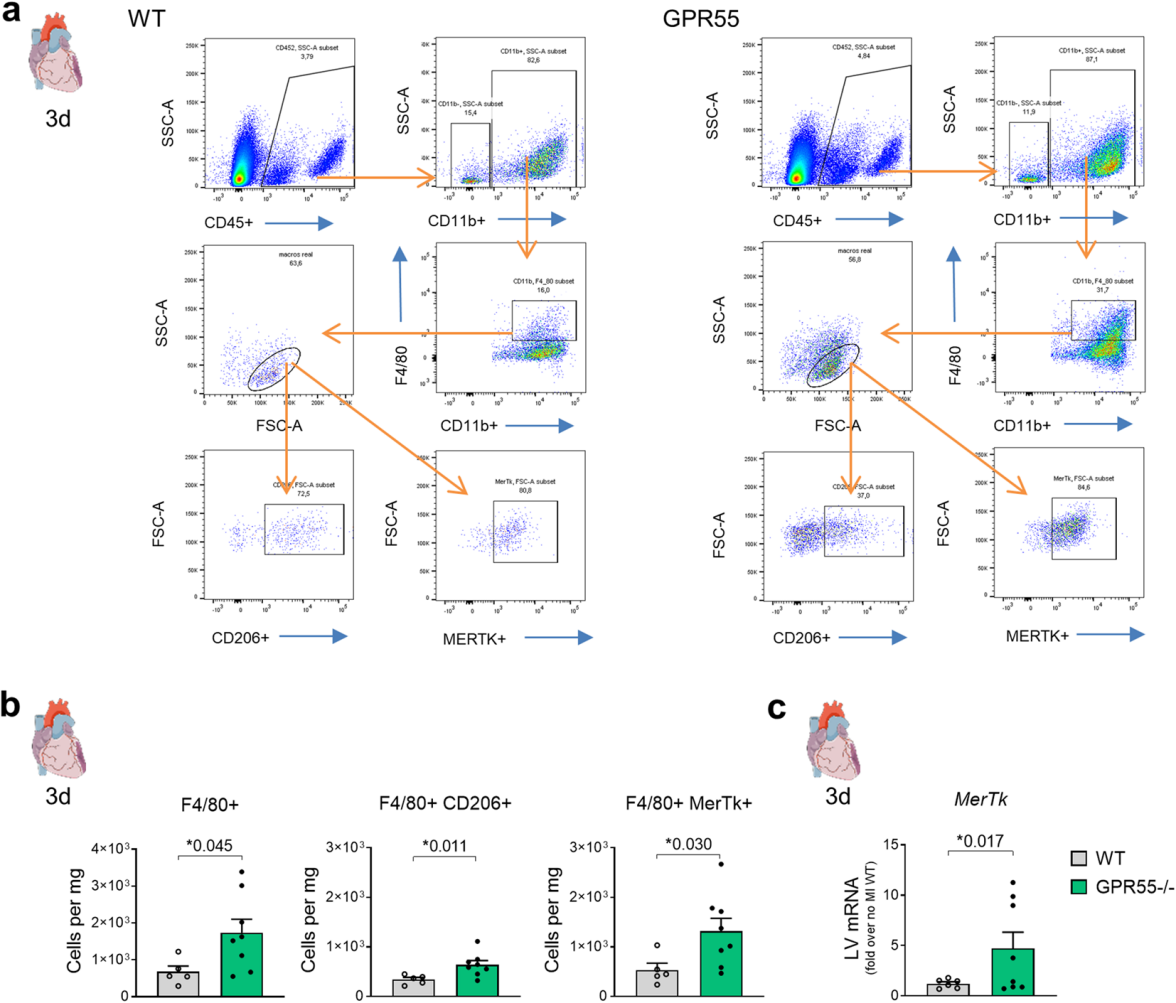
Impact of GPR55 deficiency on cardiac macrophage expansion following MI. (**a**) Representative gating for flow cytometric quantification of F4/80+ macrophages and CD206+ as well as MERTK+ populations in WT and GPR55−/− hearts. (**b**) Quantification of total macrophage counts and subsets 3 days post-MI in WT and GPR55−/− mice. (**c**) LV *Mertk* gene expression in WT and GPR55−/− mice 3 days post-MI relative to no MI WT. Bars indicate mean ± SE (n = 5–10/group) (Student’s t-test).

Despite comparable kinetics of LV neutrophil and monocyte accumulation, GPR55−/− hearts surprisingly displayed increased macrophage counts 3 days post-MI. This was reflected by concomitantly more abundant CD206+ and MERTK+ subsets when compared to WT hearts (Fig. 3a,b). Increased *Mertk* expression in GPR55 deficient heart could further be confirmed via qPCR of whole heart lysate at day 3 following MI (Fig. 3c). The mannose receptor CD206 represents a hallmark of alternatively activated, rather pro-reparative macrophages while MERTK is a phagocytosis receptor for apoptotic cells, which is crucial for tissue repair. Thus, our observations may hint toward a premature induction of reparative processes post-MI in the absence of GPR55, which is paralleled by a delayed pro-inflammatory cytokine response.

### Haematopoietic GPR55 depletion promotes cardiac expansion of pro-repair macrophages post-MI

**Figure 4:**
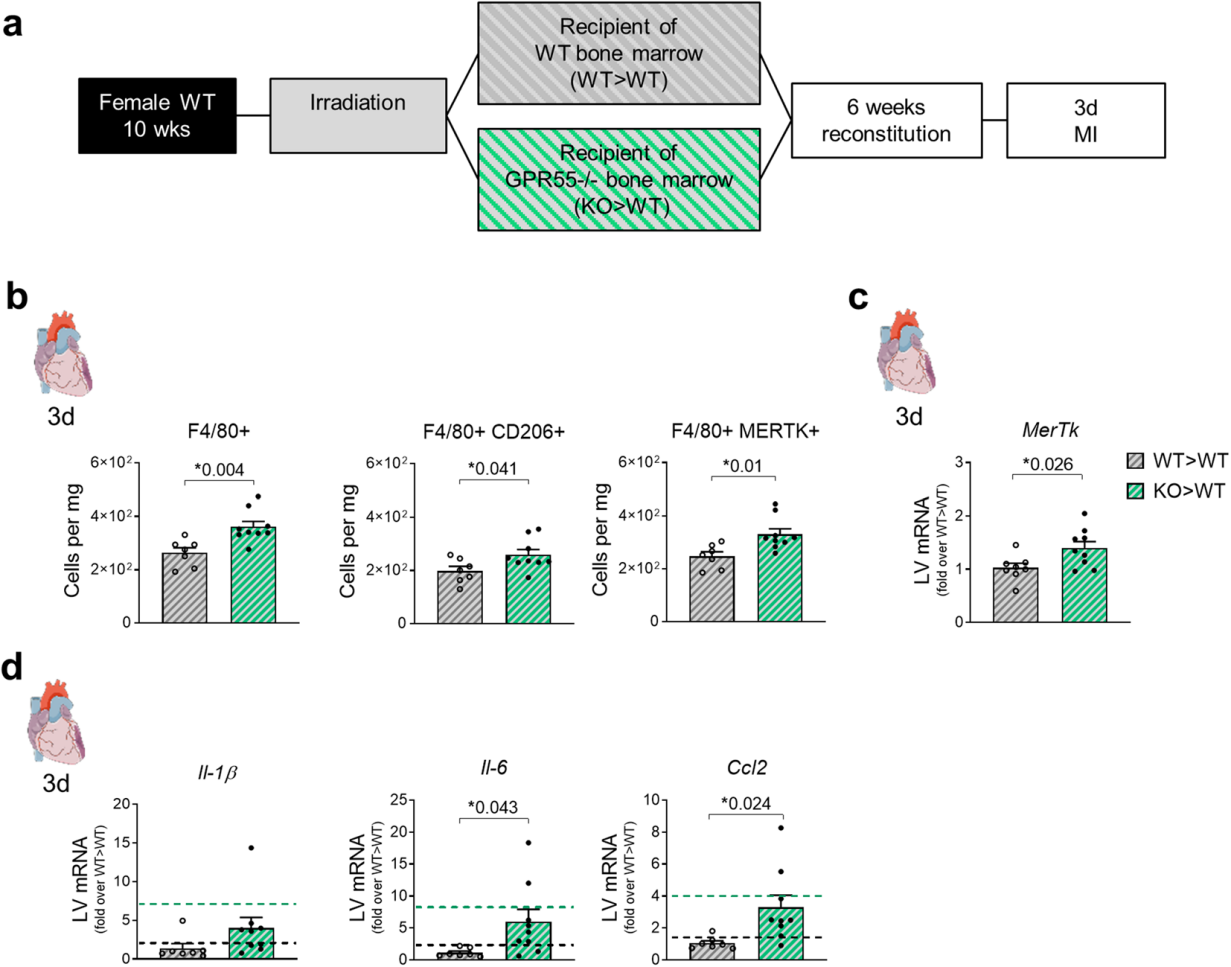
Impact of haematopoietic GPR55 depletion on cardiac macrophage expansion post-MI. (**a**) Bone marrow chimaera were generated by lethal irradiation of WT mice and subsequent reconstitution with bone marrow from GPR55−/− mice (KO > WT) or WT donors (WT > WT) as controls. After 6 weeks of recovery, both groups were subjected to 3 days MI. (**b**) Cardiac macrophage counts, and herein CD206^+^ and MERTK^+^ subsets 3 days post-MI in WT > WT and KO > WT chimaera. (**c**) LV *Mertk* gene expression in WT > WT and KO > WT chimaera 3 days post-MI, expressed as fold over WT > WT. (**d**) LV chemokine gene expression in WT > WT and KO > WT 3 days post-MI, expressed as fold over WT > WT. Dotted lines indicate transcript levels in WT and global GPR55−/− mice 3 days post-MI, as fold over WT MI. Bars indicate mean ± SE (n = 8–9/group) (Student’s t-test).

To assess the specific role of haematopoietic GPR55 in regulating cardiac macrophage expansion and polarization, we generated WT > WT and KO > WT bone marrow chimaera and subjected them to 3 days permanent MI (Fig. 4a). Sole haematopoietic depletion of GPR55 recapitulated the effects of global GPR55 deficiency regarding elevated numbers of CD206+ and MERTK+ macrophage subsets in KO > WT hearts when compared to WT > WT hearts (Fig. 4b). Moreover, increased *Mertk* transcription in 3-day infarcted KO > WT hearts could be confirmed via qPCR analyses (Fig. 4c), along with elevated cytokine and chemokine expression (Fig. 4d).

### GPR55 deficiency promotes extracellular matrix turn-over during early remodelling post-MI

**Figure 5:**
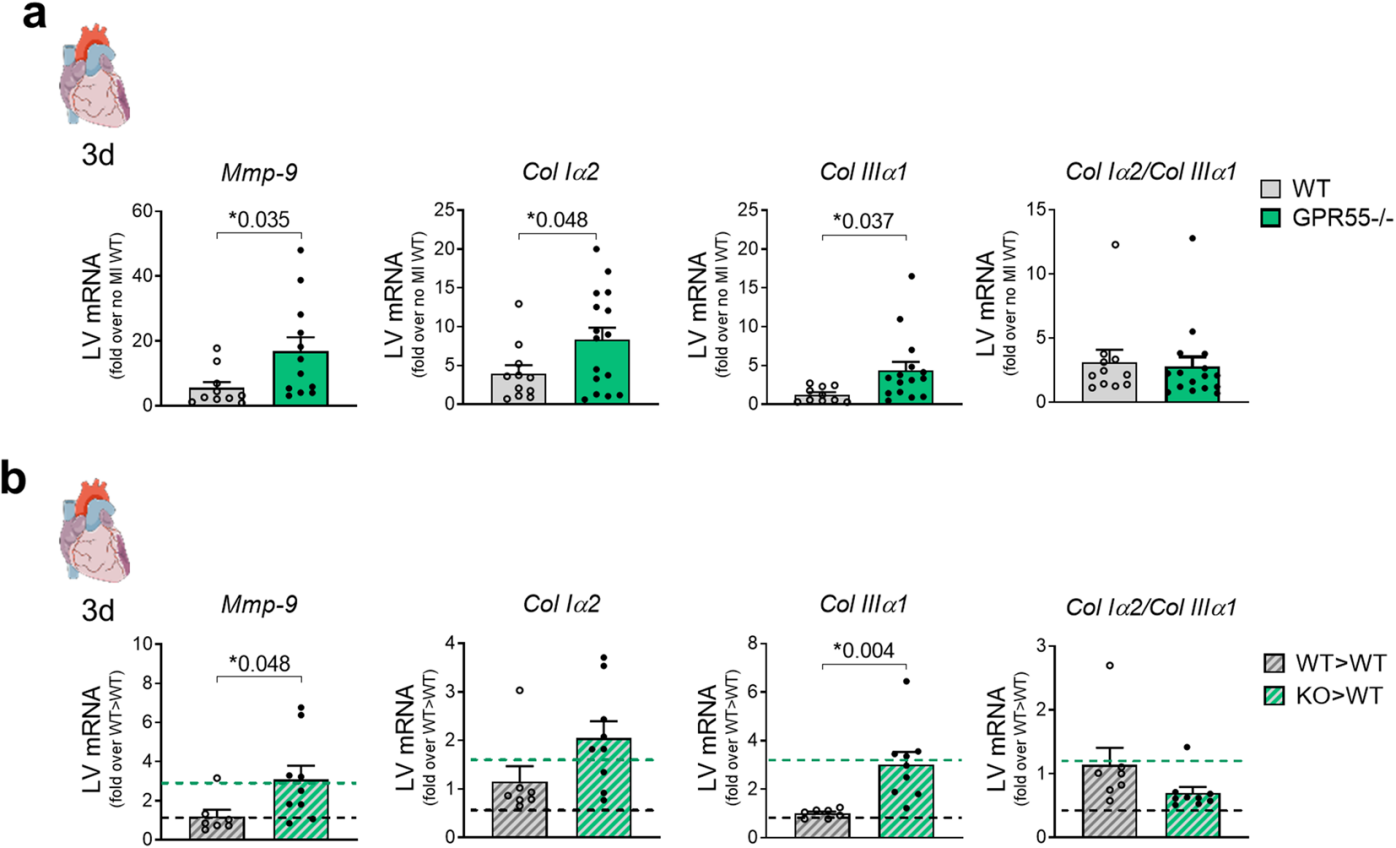
Impact of GPR55 deficiency on ECM factors following MI. LV gene expression of *Mmp-9*, *collagen Iα2*, *collagen IIIα1* and collagen variant transcript ratio 3 days post-MI in (**a**) WT and GPR55−/− mice, fold over no MI WT (n = 10–16/group) and (**b**) WT > WT and KO > WT fold over WT > WT. Dotted lines indicate transcript levels in WT and global GPR55−/− mice 3 days post-MI, as fold over WT MI. Bars indicate mean ± SE (n = 7–9/group) (Two-way ANOVA with Sidak’s post hoc test or student’s t-test, as appropriate).

Tightly regulated acute inflammation and its adequate resolution pave the way for subsequent repair mechanisms, including not only reprograming pro-inflammatory to pro-reparative macrophages, but also fibrosis for the formation of a stable scar in the infarct area. Yet, dys-regulated extracellular matrix deposition may lead to infarction expansion and LV dilatation. At day 3 post-MI, GPR55 deficiency significantly enhanced MI-induced transcription of *Mmp-9*, a potent trigger of and predictor for infarction expansion and therefore progressive maladaptation (Fig. 5a). This was accompanied by increased *Col Iα2* and *Col IIIα1* expression compared to WT mice. Ratio between *Col Iα2*/*Col IIIα2* remained unaffected by the genotype. Enhanced *Mmp-9* and collagen marker expression after MI could be mimicked by sole haematopoietic GPR55 depletion in bone marrow chimaera (Fig. 5b). These data hint toward an exaggerated matrix turnover early after the ischaemic insult and again underpin the assumption that lack of GPR55 is associated with a dysregulated healing response.

### GPR55 deficiency impairs early foetal gene expression and aggravates late structural remodelling post-MI

**Figure 6:**
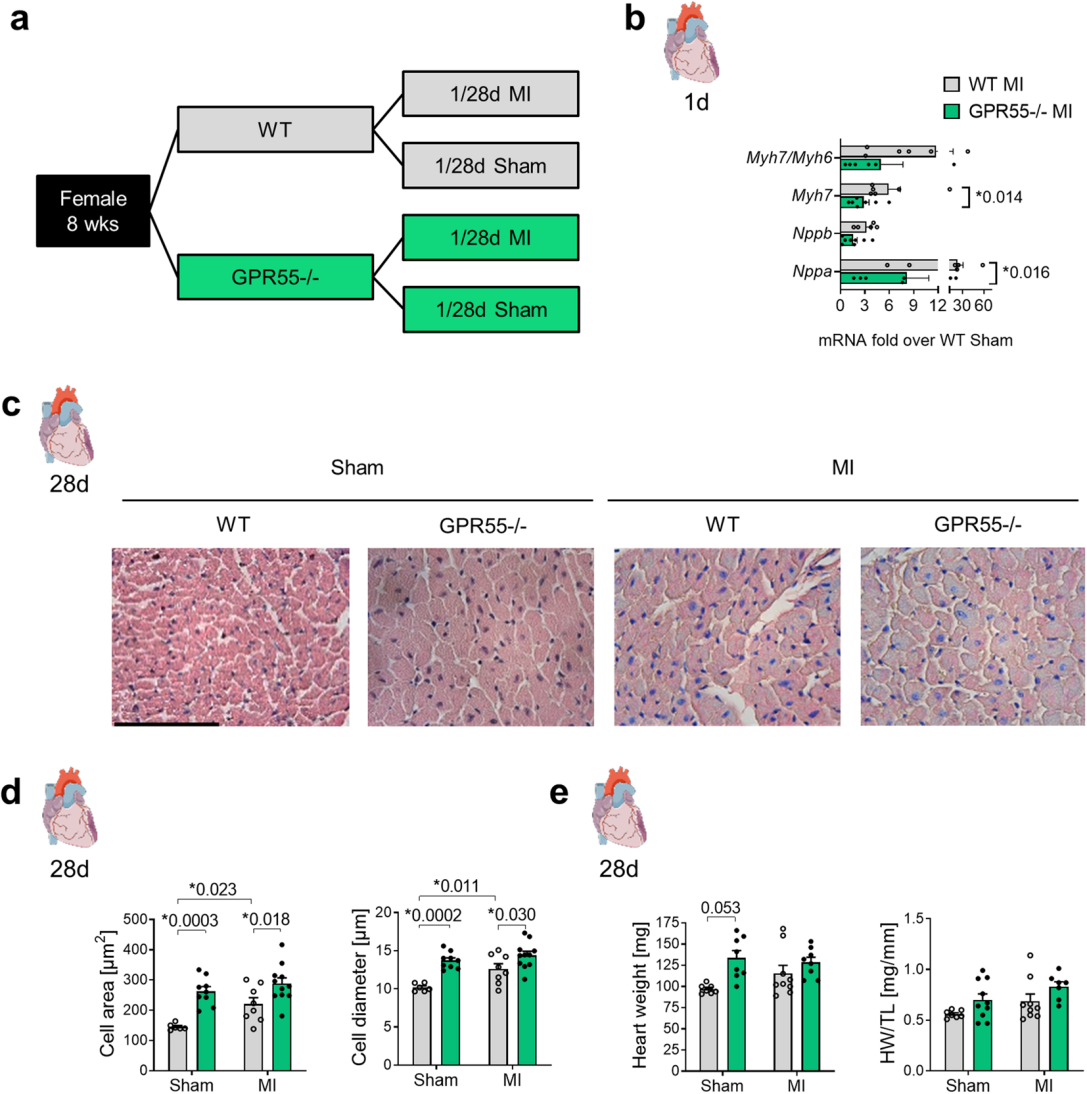
Impact of GPR55 deficiency on LV hypertrophy following MI. (**a**) Study design to assess the role of GPR55 in early hypertrophy and long-term remodelling. GPR55−/− and WT mice were randomly assigned to MI or sham surgery for 1 day or 28 days. (**b**) LV expression of foetal genes and *Myh7* expression relative to the adult MHC isoform *Myh6* in WT and GPR55−/− mice 1 day post-MI, as fold over Sham WT. (**c**) Representative haematoxylin & eosin stained midventricular cross-sections from WT and GPR55−/− mice 28 days post-MI or sham surgery, respectively (× 40 magnification, scale bar 100 µm) and (**d**) quantification of cardiomyocyte cross sectional area and diameter. (**e**) Heart weight (HW) per se and normalized against tibia length (TL) in WT and GPR55−/− mice 28 days post-MI or sham surgery, respectively. Bars indicate mean ± SE (n = 6–11/group) (Two-way ANOVA with Sidak’s post hoc test).

Not only a tightly regulated acute inflammation and matrix deposition, but also an adequate induction of compensatory hypertrophy crucially determines the outcome post-MI. To examine the impact of GPR55 on early hypertrophy and long-term remodelling post-MI, a second subset of GPR55−/− and WT mice was characterized based on hypertrophic features 1 day and 28 days post-MI or sham surgery, respectively (Fig. 6a). LV expression pattern of foetal genes revealed that GPR55 deficiency mitigated the MI triggered re-expression of *Nppa* (ANP) and *Myh7* (β-MHC) and tended to reduce *Myh7*/*Myh6* ratio, while *Nppb* (BNP) remained widely unaffected (Fig. 6b, Table 2). In contrast to the pro-inflammatory response, the pro-hypertrophic response was not delayed or shifted to a later time-point post-MI (data not shown) but rather attenuated in general.

**Table 2:** LV foetal gene re-expression 1 day after surgery in WT and GPR55 − / − mice.

	Sham		MI	
	WT	GPR55 − / −	WT	GPR55 − / −
n	6–7	7–9	5–6	6–8
*Myh7/Myh6*	1.1 ± 0.2	1.2 ± 0.4	11.8 ± 5.3*	4.9 ± 2.7
*Myh7*	1.2 ± 0.4	2.8 ± 0.7	5.9 ± 1.4	2.9 ± 0.7^#^
*Nppb*	1.8 ± 0.8	1.3 ± 0.5	3.2 ± 0.6	1.6 ± 0.5
*Nppa*	1.8 ± 0.6	2.3 ± 0.5	23.2 ± 7.7*	8.2 ± 2.7^#^

Mean ± SE; *p < 0.05 vs. corresponding Sham, ^#^p < 0.05 vs. corresponding WT, two-way ANOVA with Sidak’s post hoc test.

**Figure 7:**
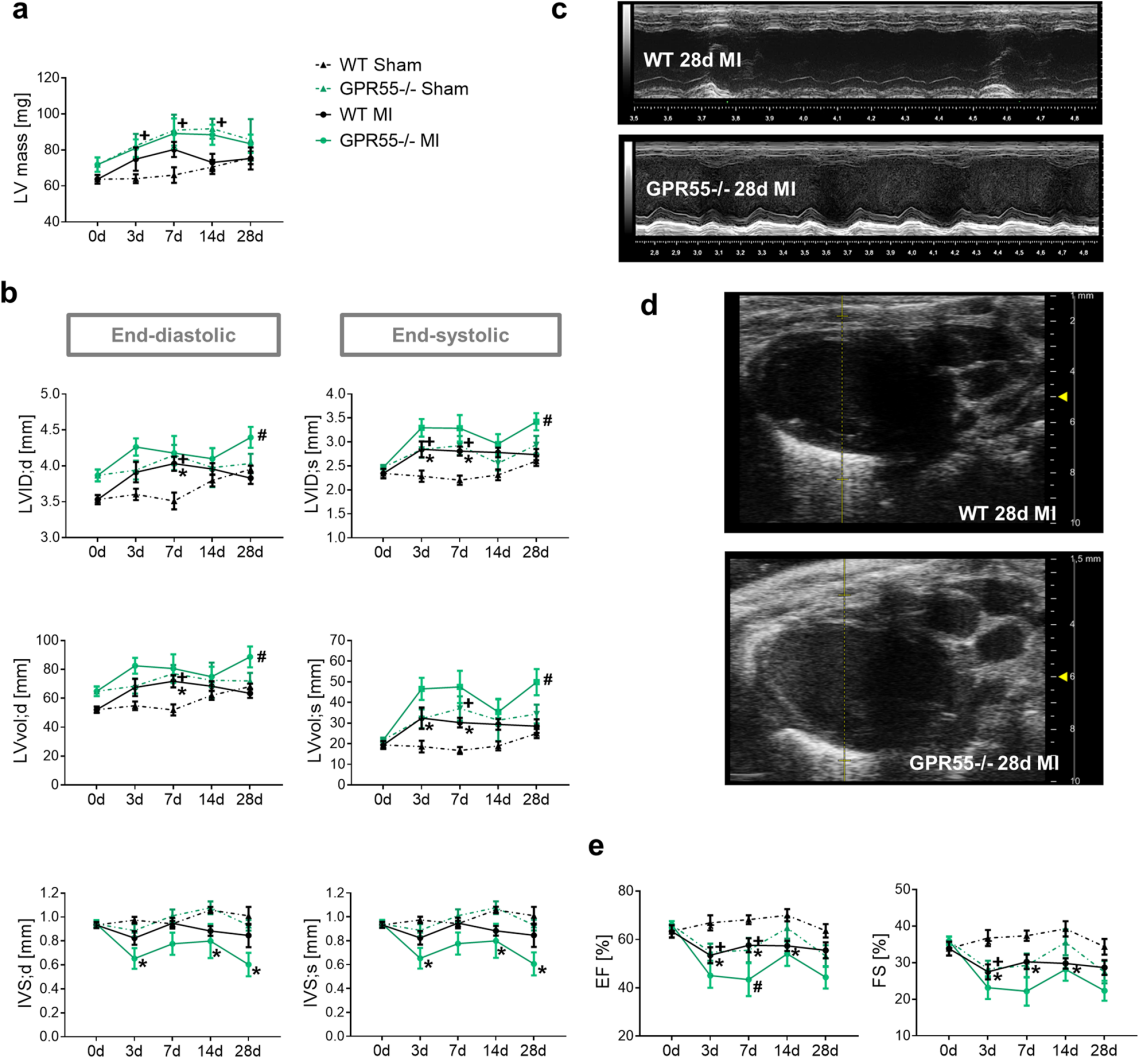
Impact of GPR55 deficiency on structural and functional maladaptive remodelling following MI. (**a**) LV mass, (**b**) diameter, volume and IVS thickness acquired by echocardiographic analyses of WT and GPR55−/− mice under basal conditions (0 day) and 3, 7, 14 and 28 days post-MI/sham surgery. (**c**) Representative long-axis M-mode and (**d**) B-mode projections at 28 day post-MI in WT and GPR55−/− mice. (**e**) LV function of WT and GPR55−/− mice under basal conditions (0 day) and 3, 7, 14 and 28 days post-MI/sham surgery. 0 day values of WT and GPR55−/− mice before MI induction are the same data as displayed in Fig. 1a and Table S1 as baseline data. Circles and triangles indicate mean ± SE (n = 7–14/group). *p < 0.05 vs. corresponding Sham, ^#^p < 0.05 vs. corresponding WT; ^+^p < 0.05 GPR55−/− Sham vs. WT Sham (Two-way ANOVA with Sidak’s post hoc test).

Measurement of cardiomyocyte cross sectional area and diameter confirmed MI triggered hypertrophy in WTs in comparison to WT sham mice (Fig. 6c,d). Interestingly, independent of the surgical intervention, GPR55−/− mice exhibited larger cardiomyocytes than their corresponding WT groups, with no evident enlargement by MI. A similar trend could be observed for heart weight per se and normalized against tibia length and body weight (Fig. 6e, see Supplementary Table S2). Consistent with this observation, echocardiography could not reveal an additive effect of MI on the pre-existing higher LV mass in GPR55−/− mice under sham-conditions (Fig. 7a). Furthermore, the enlarged end-diastolic diameter and volume, which was already observed under naïve conditions (Fig. 1a), was also noted at early time-points after sham surgery, accompanied by an early increase in end-systolic chamber size and filling. This difference between the sham groups was blunted by physiological age-dependent increase in WT heart size from day 7 onwards. These observations most likely count for first, the lack of evident MI triggered LV dilatation in GPR55−/− mice during the first 7 days post MI and second the pronounced dilatation seen in WTs during this remodelling stage when compared to their respective sham groups. Nonetheless, GPR55 deficiency significantly aggravated post-MI LV dilatation during the late remodelling phase (Fig. 7b–d). While lack of GPR55 per se did not affect LV wall thickness over time in the sham group compared to WT sham, post-MI maladaptation in GPR55−/− mice was additionally characterized by a drastic and early thinning of both, the LV anterior wall (LVAW) and the intraventricular septum (IVS), hinting toward aggravated infarction expansion (Fig. 7b,c, see Supplementary Fig. S5). Of note, while MI expectedly reduced EF and FS in WTs compared to their sham controls, we also observed a reduced LV function in GPR55−/− mice early after sham-surgery as consequence of the increased end-systolic volume. Nevertheless, MI triggered a further functional decline in GPR55 deficient mice, as verified by lower EF in comparison to the WT MI group, which was most pronounced at 7 days post MI (Fig. 7e).

## Discussion

We provide novel evidence that the lipid receptor GPR55 crucially affects the heart during homeostasis and post-MI pathophysiology. Our data hint toward a relevant role for GPR55 signalling in both, haematopoietic cells and cardiomyocytes in regulating cardiac function and remodelling. Baseline phenotyping suggests GPR55 to affect preload and chronotropy. Increase in end-diastolic LV volume and stroke volume presumably compensate for the reduced heart rate to maintain cardiac output in GPR55−/− mice. Of note, our observation that GPR55−/− mice exhibit low heart rates (367 ± 14 bpm), compared to WT (515 ± 5 bpm and an average EF of 63%), initially derived from echocardiographic recordings under 1.5% isoflurane inhalation. In contrast, Walsh et al. reported, under ketamine/xylazine anaesthesia during catheterization, an increased HR in naïve GPR55−/− mice (420 ± 7 bpm) compared to WT, with the latter exhibiting a rather low frequency of merely 395 ± 7 bpm, associated with an EF of only 50%^25^. It is conceivable that the distinct examination techniques (non-invasive vs. invasive) and/or the differing HR suppressing impact of the applied anaesthetics might account for the divergent observations between the two studies. However, heart rate recordings after isoflurane withdrawal in conscious mice confirmed GPR55 to affect chronotropy widely independent of an anaesthesia interaction in our study (WT 564 ± 10 bpm vs. GPR55−/− 366 ± 13 bpm). GPR55 deficiency might generate the negative chronotropy by dampening neurotransmitter release and reduced adrenergic signalling, while GPR55 stimulation with LPI increases transmitter exocytosis, as shown in PC12 cells^30^.

The reason for increased diastolic volume and diameter in GPR55−/− mice in the absence of evident valvular defects could be twofold: First, a pre-established increase in blood volume and excessive fluid accumulation in the body; Second, impaired lusitropy and/or a prolonged filling phase creating a pre-stretch, maybe resulting from altered ion sensitivity. Similar heart, atria and lung weight to body weight ratios, as well as comparable plasma albumin level and plasma volumes rather rule out an excessive fluid accumulation in GPR55−/− mice as cause for the enhanced LV volume^31–33^. In contrast, after verifying GPR55 expression in adult murine cardiomyocytes, we observed an enhanced diastolic, but not systolic sarcomere length in GPR55−/− cardiomyocytes compared to WT myocytes. This was accompanied by an accelerated relaxation time and increased Ca^2+^ transient amplitude. Altered Ca^2+^ entry and intracellular Ca^2+^ mobilization in response to GPR55 stimulation with cannabinoids or LPI has been shown in isolated neonatal rat cardiomyocytes^24^. Moreover, transient expression of human GPR55 in HEK293 cells increases intracellular [Ca^2+^] and inhibits potassium M currents^34^. GPR55 deficiency might consequently modify lusitropy, diastolic filling and chronotropy by altering not only Ca^2+^ handling but also K^+^ efflux. Our data confirm for the first time a direct function of the receptor in adult murine cardiomyocytes and underpin the hypothesis that lack of the receptor manifests a pre-stretch of the left ventricle. We suppose GPR55 to regulate filling dynamics, thereby mediating LV volume-overload and wall stretch by modifying myofilament sensitivity. Moreover, we might speculate GPR55 deficiency not only to promote LV pre-stretch, but also to affect action potential duration and action potential rate, i.e. heart rate by altering ion handling and filament sensitivity.

Permanent LV volume-overload applies a constant mechanical wall stress, forcing cardiomyocytes to adapt by enlargement and inducing moderate myocardial inflammation triggering LV neutrophil infiltration, regulated by *Tnfα* and *Ccl2*, resulting in MMP activation^35^. Moreover, enhanced dilation-triggered wall tension in vivo as well as applied mechanical stretch in vitro promotes macrophage proliferation^36^. Thus, cardiomyocyte enlargement, elevated LV counts of macrophages and neutrophils—both known to express and secrete MMP-9, together with up-regulated *Ccl2* and *Mmp-9* transcription in unstressed GPR55−/− mice may support our hypothesis of a volume-overload phenotype. GPR55−/− mice also exhibited higher femoral, splenic (not shown), circulating and cardiac neutrophil numbers and elevated plasma MPO level when compared to basal WTs, underpinning augmented neutrophil activity. Our data suggest for the first time, that functional GPR55 signalling is required for the maintenance of physiological immune homeostasis in conditions where the heart is exposed to moderate mechanical stretch. We therefore propose that GPR55 exerts direct cardiac effects, which in turn, might affect the immune system even under naïve conditions.

Following acute myocardial infarction, GPR55 deficiency did not affect the initial infarct size in our study. The literature states conflicting results regarding the impact of GPR55 on infarct size. However, these results mainly emerge from ischaemia/reperfusion (I/R) studies^26,28,37^. The controversy might also be related to the fact that ligands used to activate or antagonize GPR55 are targeting additional receptors, implying that the receptor responsible for the reported infarct size alterations is not entirely clear. Despite comparable infarct sizes in our study, GPR55−/− deficiency shifted the onset of acute inflammation from day 1 to day 3 at the site of injury, which is attributable to haematopoietic GPR55 expression. Somewhat surprising, this delayed and exacerbated pro-inflammatory response did not alter LV infiltration kinetics of monocytes and neutrophils. Balenga et al. suggested that a CB2-GPR55 cross-talk is essential for neutrophil migration to the site of injury, based on their in vitro migration assays using human blood neutrophils^23^. Our data do not support an impaired neutrophil recruitment in absence of GPR55, at least not in the context of permanent LAD ligation in mice. While GPR55 depletion had no effect on myeloid cell infiltration into the infarcted heart, it promoted expansion of alternatively activated macrophages at day 3 post-MI, the time-point when Ly6C^high^ monocyte derived rather pro-inflammatory macrophages are described to accumulate following ischaemia^10,38^. The observed expansion of CD206+ macrophages in GPR55−/− mice was associated with enhanced MERTK expression, a phagocytosis receptor that plays a crucial role in cardiac healing post-MI^39^. Haematopoietic depletion of GPR55 could mimick the macrophage expansion in the heart seen in global GPR55 knockout mice. The higher abundance of pro-reparative macrophages, together with elevated *Mmp-9* and *Col* levels in absence of GPR55 hint toward a role of the receptor in regulating matrix turn-over and macrophage polarization. Blocked GPR55 signalling has already been linked to enhanced MMP-9 expression under inflammatory conditions^40^. The molecular mechanisms polarizing macrophages toward a pro-fibrotic phenotype and thereby promoting premature wound healing, however, remain to be further investigated. We may only speculate that a disturbed Ca^2+^ signalling in cardiac macrophages in absence of GPR55 may promote their polarization to an alternative phenotype. So far, studies addressing an immuno-modulatory role for GPR55 have mainly focused on proliferation, lymphocyte function and neutrophils in haematopoietic and peripheral organs in chronic disease settings^41–43^. Our data indicate for the first time a GPR55-dependent regulation of macrophage expansion and phenotype in the heart. In support of a role for GPR55 in cardiac macrophages, we confirmed its gene expression in macrophages sorted from naïve hearts. We may speculate that GPR55 is also expressed by de novo recruited bone-marrow derived macrophages to the infarcted myocardium, although we did not directly confirm this due to the lack of specific antibodies for flow cytometric detection within ischaemic cardiac tissue.

In addition to the haematopoietic role of GPR55 during MI healing, we also observed an impact of the receptor on cardiomyocyte morphology and signalling. GPR55−/− mice exhibited enlarged cardiomyocytes under sham conditions. A further MI-triggered increase in myocyte size, i.e. pathological hypertrophy, was blunted in the absence of GPR55. In support of a crucial role for GPR55 in regulating hypertrophic responses, we also noticed an attenuated re-activation of foetal genes in GPR55−/− hearts. The reported dysregulated healing response, encompassing premature LV expansion of pro-reparative macrophages combined with increased transcription of ECM factors and prolonged exacerbated chemokine production could be clearly attributed to a haematopoietic role of GPR55. Aggravated long-term remodelling, including exacerbated LV dilatation, infarct thinning and expansion and a more pronounced functional decline, most likely derives from a combination of the haematopoietic impact, mitigated compensatory cardiomyocyte hypertrophy and the basal volume-overloaded cardiac phenotype in the absence of GPR55. In addition, it is quite conceivable that dysregulated glucose uptake and insulin secretion may contribute to the harmful effects of GPR55 deficiency on post-MI outcome. Given that GPR55 activation reduces glucose levels, stimulates insulin secretion from pancreatic β cells, promotes insulin signalling in myotubes, and contributes to manifestation of obesity and diabetes, the receptor could also impact on the metabolically imbalanced myocardium upon ischaemic injury^20,21,44^. Herein, the receptor might exert peripheral effects (substrate supply) or direct cardiomyocyte effects by affecting glucose and fatty acid up-take and utilization. This however needs to be elucidated by further cardiac studies. In conclusion, our study alludes for the first time toward a crucial role for GPR55 in regulating heart rate, maintaining adequate LV load and stretch, inducing compensatory hypertrophy, synchronizing wound healing and limiting maladaptation post-MI via synergistic cardiac and haematopoietic effects. Thus, targeted GPR55 stimulation might beneficially impact on patient’s clinical outcome post-MI (for a graphical summary see Supplementary Fig. S6).

Finally, we realize that our study has several limitations. First, in this study we applied permanent LAD ligation to induce MI. Even though I/R models are assessed more clinically relevant, the widely used permanent LAD occlusion allows utter tracing of the wound healing and remodelling effects elicited by genetic, pharmacological, or surgical interventions. This in turn can provide insights into the role of specific cell types, signalling cascades and structural alterations triggered by ischaemia, preceding more defined therapeutically relevant approaches. Second, at this stage we cannot exclude that the global constitutive GPR55 deficiency provokes compensatory mechanisms, such as the up-regulation of other cannabinoid-sensitive lipid receptors or lipid mediator synthesizing and degrading enzymes. Targeted GPR55 blockade and/or stimulation approaches as well as cell-specific receptor depletion studies are warranted to further decipher the exact role of the receptor in post-MI wound healing. Third, we suggest GPR55 to contribute to the development of LV volume overload. Further studies investigating the role of GPR55 in experimental overload settings are required to underpin our assumptions.

## Methods

### Study population

GPR55−/− mice on C57BL/6J background were purchased from the European Mouse Mutant Archive (EM:02355). The targeted mutation was generated in 129SvEvBrd-derived embryonic stem cells via insertion of a targeting cassette depleting the entire GPR55 encoding sequence in exon 1 (International strain designation: B6; 129S5-Gpr55tm1Lex/leg) and back-crossing of offspring with C57BL/6J WT mice. WT littermates were obtained by heterozygous breeding and served as controls throughout the study. Mice were genotyped after weaning by PCR to amplify a 422 bp region of the WT and a 301 bp region of the mutant allele. WT and GPR55−/− female mice were included in this study. No signs of infertility, pre-mature mortality, developmental defects or sex-specific differences in basal phenotypes were observed in a pilot study. All animal procedures were performed conform to the guidelines from Directive 2010/63/EU and the ARRIVE guidelines, and approved by the government of Upper Bavaria (ROB-55.2.2532.Vet_02-13-176 and 18-114).

### Echocardiography

Echocardiographic scans were acquired with the VisualSonicsVevo^®^ 3100 imaging system (Scanhead: RMV707B, 15–45 MHz, cardiac mouse) and analysed as described previously by Puhl et al.^45^. For serial scans mice were basally scanned 2 to 3 days before surgery and 3, 7, 14 and 28 days post-intervention. To assess heart rate under conscious conditions, mice were initially anaesthetized and monitored as described above and ECG recordings were continued upon isoflurane withdrawal and regaining consciousness.

### MI induction

GPR55−/− and WT mice were randomly assigned to either permanent ligation of the left anterior descending coronary artery (LAD) using a 7-0 thread to induce MI or to sham operation, respectively. Briefly, under deep anaesthesia (medetomidin/midazolam/fentanyl; 0.5/5.0/0.05 mg/kg; intraperitoneally) the thorax was opened in the third intercostal room and the LAD was ligated 2 mm below the left atrium. The intercostal space and skin were closed with single knot sutures with 5-0 silk. Anaesthesia was antagonized by flumazenil/atipamezol (0.1/0.5 mg/kg; intraperitoneally). For analgesia buprenorphine (0.05 mg/kg body weight; subcutaneously) was injected immediately, 6 h, 24 h and 32 h and meloxicam (0.2 mg/kg; subcutaneously) 24 h after surgery. Sham animals underwent the same procedure without occlusion of the LAD.

### Bone marrow transplantation

Bone marrow chimaera were generated by lethal irradiation (5 Gy; twice within 8 h) of female WT mice, aged 10 weeks, and subsequent reconstitution with 2 × 10^6^ bone marrow cells extracted from femurs of female, age matched global GPR55−/− mice (KO > WT) or WT donors (WT > WT). Mice received antibiosis (100 µg/ml neomycin and 10 µg/ml polymyxin B sulphate) via drinking water starting 1 week before and continuing 4 weeks after irradiation and bone marrow transplantation. After a recovery phase of 6 weeks following reconstitution, mice were subjected to permanent LAD ligation for 3 days.

### Tissue sampling

Mice were euthanized by overdosed xylazine/ketamine (120/10 mg/kg body weight) anaesthesia. EDTA blood was collected from the right ventricle and split for flow cytometry (50 µl) and plasma collection. After LV puncture and perfusion with PBS, the heart, lungs, spleen, liver, left kidney, femoral bone marrow and left tibia were excised. Wet and dry weight of lungs, liver and the left kidney were determined to assess tissue congestion. A description of tissue processing and LV partition for different analyses can be found in Supplementary Methods and Supplementary Fig. S7.

### Flow cytometry

Bone marrow cells were collected from one femur by centrifugation for 2 min at 9000×*g*. Excised spleens were mashed through a 70 µm filter. EDTA blood, bone marrow cells and spleens were subjected to erythrolysis. Hearts were minced with fine scissors and digested, as described previously^19^. The entire list of antibodies used and the applied gating strategies can be found in Supplementary Methods and the Supplementary Fig. S8.

### Histology

Cardiomyocyte cross-sectional area and diameter were assessed using the LEICA LAS software following staining with haematoxylin and eosin (H&E) in paraformaldehyde-fixed and paraffin-embedded transverse midventricular tissue slices (5 µm), as described previously^46^.

### Gene expression analyses

For qPCR analysis of whole cell lysates, the respective tissue was homogenised, lysed and RNA isolated. Following reverse transcription 2.3 ng cDNA per sample and gene were used for TaqMan PCR amplification. All samples were run in duplicates. mRNA expression was normalized per sample against *Gapdh* and relative quantitation of gene expression was calculated using the comparative ∆∆CT method. Expression data based on whole tissue mRNA are displayed as fold over WT no MI, WT Sham or WT > WT, as appropriate. The entire list of qPCR primers used can be found in Supplementary Methods.

### ELISA

Plasma MPO levels were determined using a standard ELISA kit (Duoset, R&D Systems).

### Sarcomere length and Ca^2+^ transient measurement

LV myocytes were isolated from adult WT and GPR55−/− mice with an isolation protocol modified from O’Connell et al. (AfCS Research Reports; Vol. 1 No. 5 CM) according to Nickel et al.^47,48^ and Kohlhaas et al.^49^. Isolated cardiomyocytes were paced by electrical field stimulation and sarcomere length was recorded using a customized IonOptix system as described previously^47,49^. [Ca^2+^]c was measured by incubating cells with indo-1 AM (5 µmol/l) for 20 min at 25 °C (λexcitation = 340 nm, λemission = 405/485 nm).

### Data analysis

Experimental animals were randomly assigned to treatment groups. Group sizes throughout the study are n ≥ 5. Continuous data are presented as mean ± SE. To determine differences between two groups, unpaired student’s t-test was performed. For multiple group comparisons involving two independent variables and for parallel repeated measures to determine the significance for individual time points, two-way ANOVA was applied with Sidak’s post-hoc test after F-test of variance was confirmed as p < 0.05. For repeated (paired) Ca^2+^ and sarcomere length measurements two-way ANOVA with Bonferroni’s post hoc test was applied. All calculated p-values are two-sided. Differences with a p < 0.05 were considered statistically significant. All analyses were performed with Microsoft Excel 2010 and GraphPad Prism 8.0 software.

### Ethics approval

All animal procedures were performed conform to the ARRIVE guidelines, Directive 2010/63/EU and to the government of Upper Bavaria (ROB-55.2.2532.Vet_02-13-176 and 18-114).

## Supplementary Information

Supplementary Information.

## Data Availability

The data underlying this article are available in the article and in its online supplementary material.
