# Supplementary material for: Haematopoietic and cardiac GPR55 synchronize post-myocardial infarction remodelling
Source: Sci Rep. 2021 Jul 13;11:14385. doi: 10.1038/s41598-021-93755-y (PMC8277802; doi:10.1038/s41598-021-93755-y)
Supplement: Supplementary file 1 — Supplementary Information. [file 41598_2021_93755_MOESM1_ESM.pdf]

Supplementary Information

Haematopoietic and cardiac GPR55 synchronize post-myocardial infarction remodelling

Sarah-Lena Puhl[1], Michael Hilby[1], Michael Kohlhaas[3], Linus M. Keidel[1], Yvonne Jansen[1], Michael Hristov[1], Jakob Schindler[1], Christoph Maack[3], *Sabine Steffens[1,2]

[1]Institute for Cardiovascular Prevention (IPEK), Ludwig-Maximilians-Universität (LMU) Munich, Munich, Germany

[2]German Centre for Cardiovascular Research (DZHK), partner site Munich Heart Alliance, Munich, Germany

[3]Comprehensive Heart Failure Center, University Clinic Würzburg, Würzburg, Germany

Correspondence:
Sabine.Steffens@med.uni-muenchen.de
Institute for Cardiovascular Prevention, Pettenkoferstr. 9, 80336 Munich, Germany
Phone: +49 89 4400-54674

Content

-Supplementary Figures

-Supplementary Tables

-Supplementary Methods

Supplementary Figures

Fig. S1

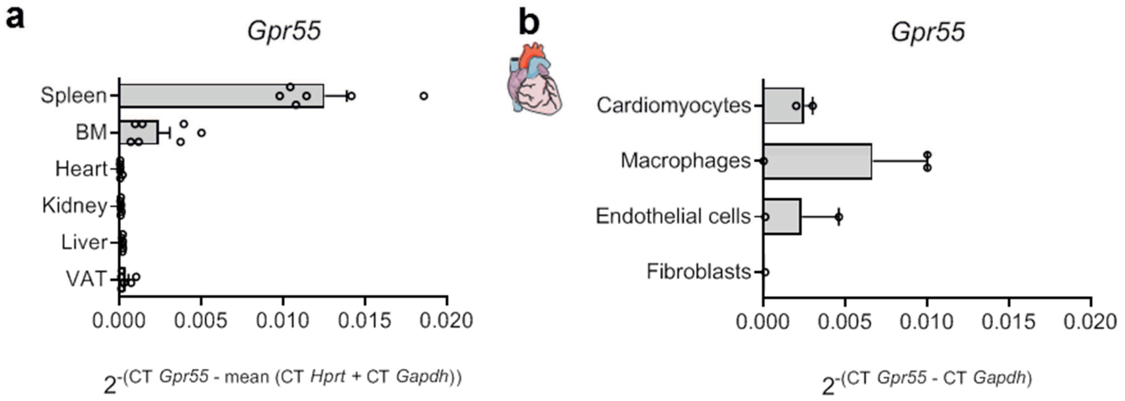

Fig. S1 Organ and cardiac cell type specific *Gpr55* expression
GPR55 mRNA expression in WT a) spleen, BM, heart, kidney, liver and VAT expressed as $2^{-(CT\ Gpr55\ -\ mean\ (CT\ Hprt\ +\ CT\ Gapdh))}$ and b) sorted cardiac myocytes, macrophages, endothelial cells and fibroblasts expressed as $2^{-(CT\ Gpr55\ -\ CT\ Gapdh)}$. BM, bone marrow; VAT, perigonadal visceral adipose tissue.

Fig. S2

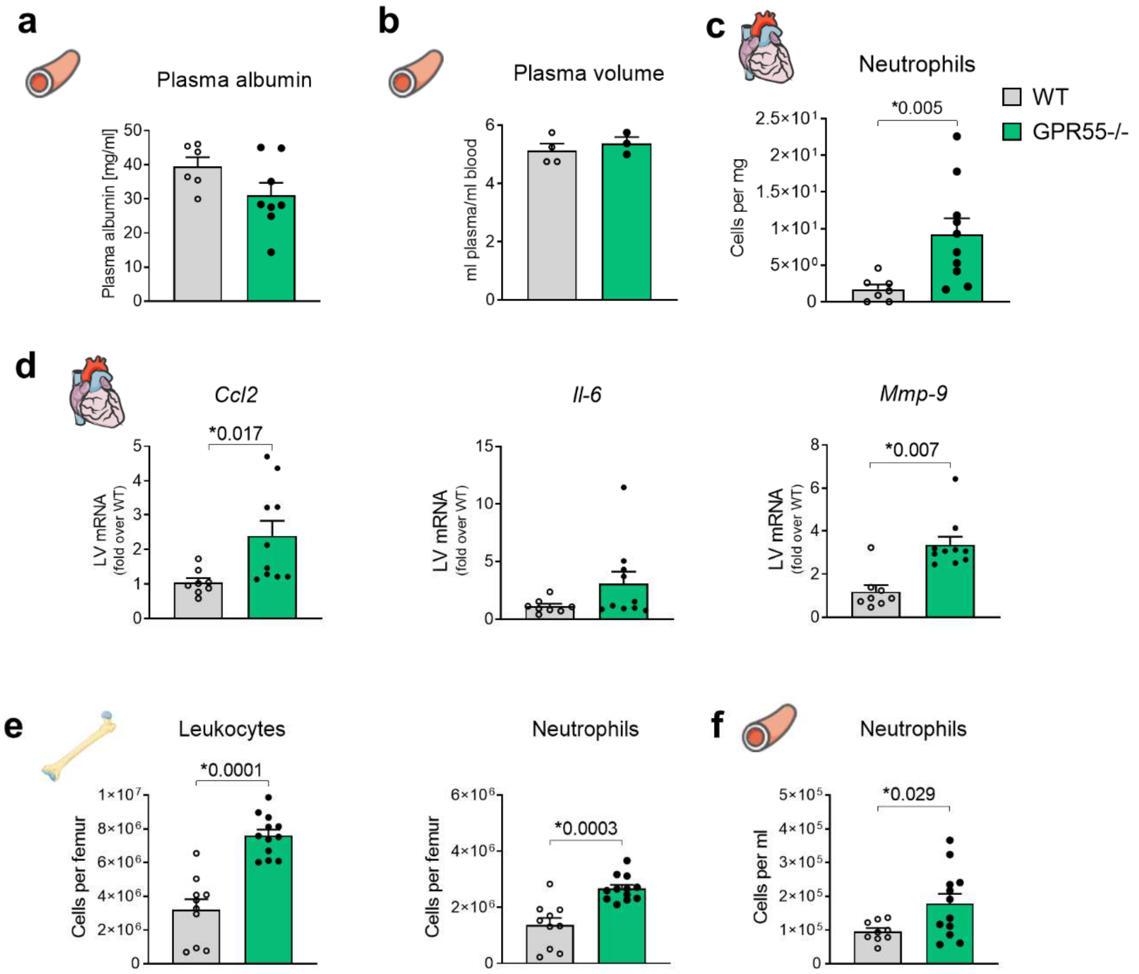

Fig. S2 Impact of GPR55 deficiency on reported markers for LV volume-overload
a) Plasma albumin level (n=6-8/group) and b) plasma volume per ml EDTA blood (n=3/group; preliminary data) in naïve WT and GPR55-/- mice. c) Basal LV neutrophil counts in WT and GPR55-/- mice, detected via flow cytometry (n=7-11/group). d) Basal LV mRNA expression of the pro-inflammatory chemokines *Ccl2* and *Il-6* and the ECM factor *Mmp-9* (n=8-9/group). e) Basal femoral leukocyte counts and herein neutrophil numbers and f) circulating neutrophil numbers in WT and GPR55-/- mice, detected via flow cytometry (n=10-12/group). Bars indicate mean±SE (Student's t-test).

Fig. S3

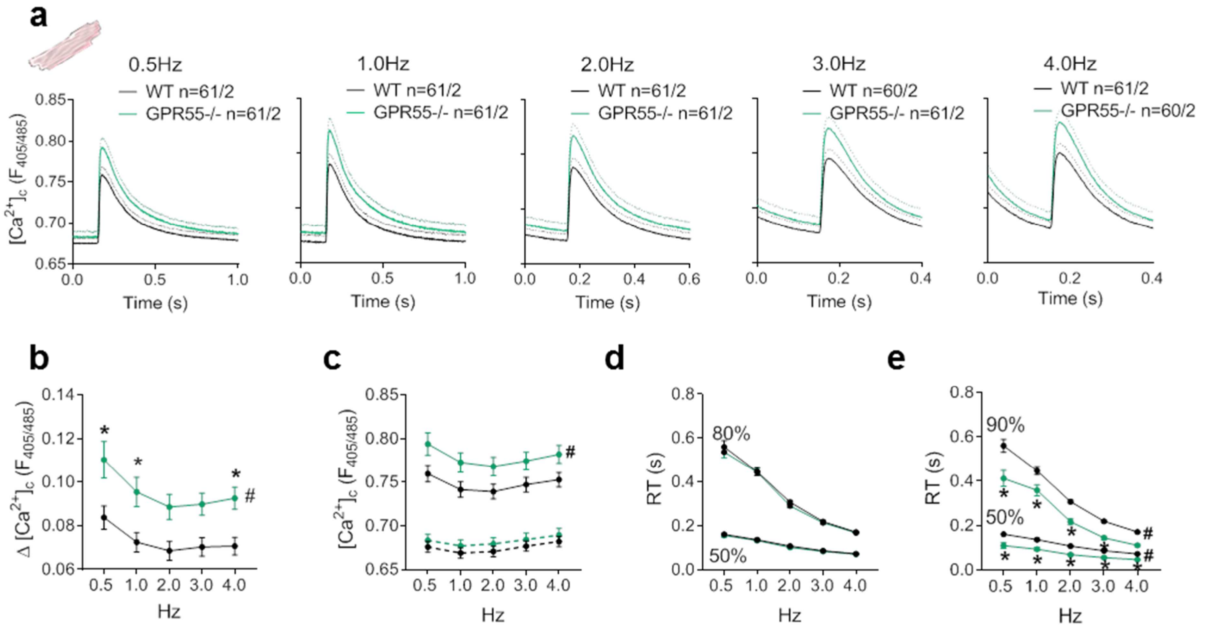

Fig. S3 Impact of GPR55 on myocyte Ca^{2+} transient

a) Averaged Ca^{2+} transients±SE (dotted lines) and b) Ca^{2+} transient amplitude of isolated WT and GPR55-/- cardiomyocytes under increasing pacing frequencies. c) Intracellular Ca^{2+} concentration of isolated WT and GPR55-/- cardiomyocytes under increasing pacing frequencies during systole (solid lines) and diastole (dotted lines). d) Time from peak tension to 50% and 80% Ca^{2+} decline (RT) and e) time from peak contraction to 50% and 90% relaxation (RT) under increasing pacing frequencies. $[Ca^{2+}]c$ was measured by incubating cells with indo-1 AM (5 µmol/L) for 20 min at 25°C (λexcitation=340 nm, λemission=405/485 nm). Circles indicate mean ±SEM; #p<0.05, two-way ANOVA with Bonferroni's post hoc test (*p<0.05 vs. WT); n indicates number of isolated cardiomyocytes from 2 mice.

Fig. S4

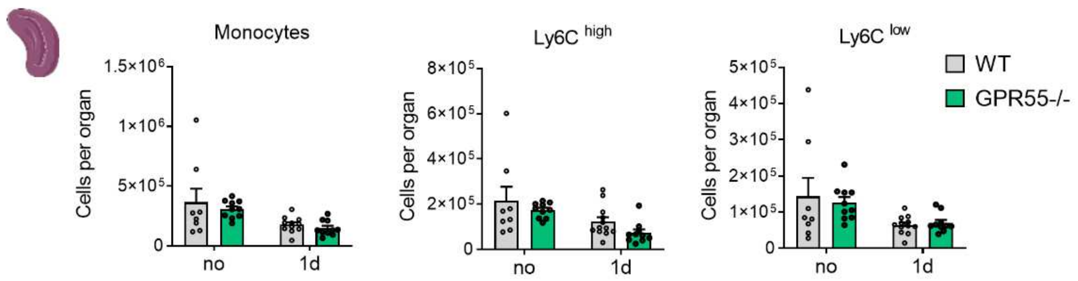

Fig. S4 Impact of GPR55 on splenic monocyte counts after acute MI

Total splenic counts of monocytes and herein $Ly6C^{high}$ and $Ly6C^{low}$ sub-populations in WT and GPR55-/- mice at baseline (no) and 1d post-MI. Bars indicate mean±SE (n=8-11/group; two-way ANOVA with Sidak's post hoc test).

Fig. S5

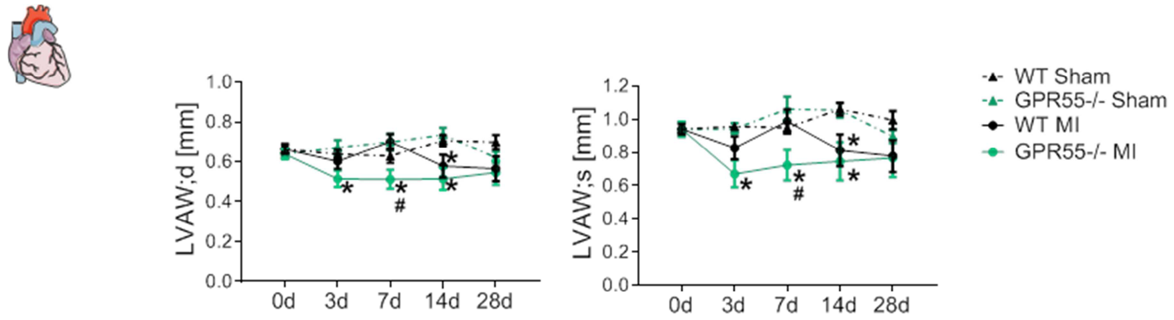

Fig. S5 Impact of GPR55 deficiency on structural maladaptive remodelling following MI
LVAW thickness acquired by echocardiographic analyses of WT and GPR55-/- mice under basal conditions (0d) and 3, 7, 14 and 28d post-MI/sham surgery. 0d values of WT and GPR55-/- mice before MI induction are displayed in Fig. 1 c and Table S1 as baseline data. Circles and triangles indicate mean±SE (n=7-14/group). *p<0.05 vs. corresponding Sham, #p<0.05 vs. corresponding WT; +p<0.05 GPR55-/- sham vs. WT sham (two-way ANOVA with Sidak's post hoc test).

Fig. S6

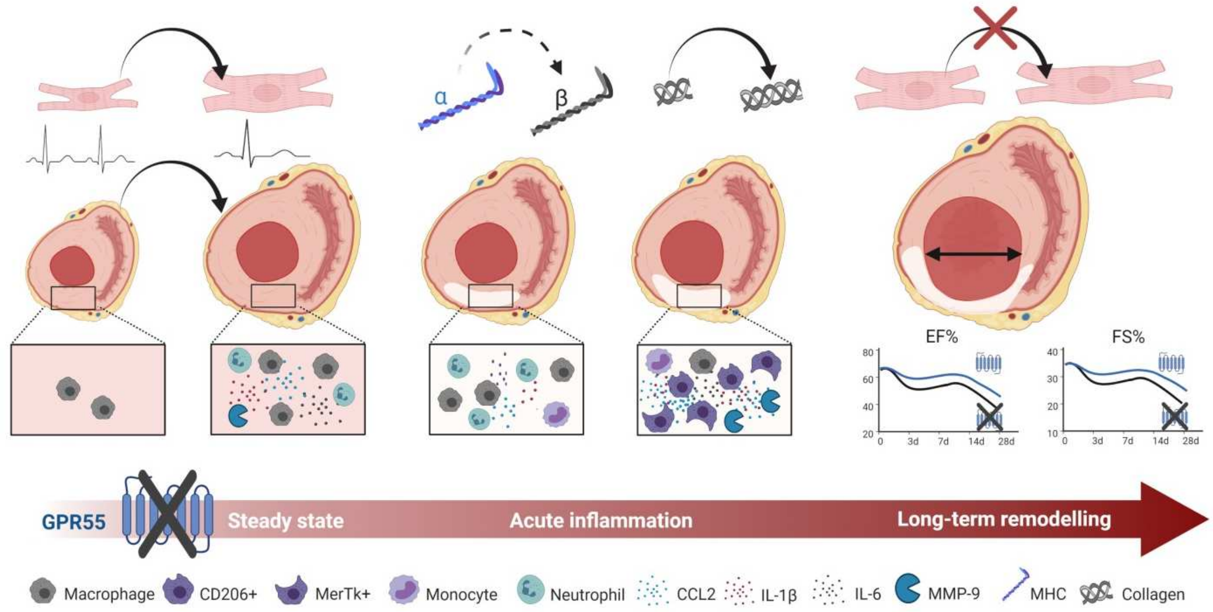

Fig. S6 Summary of experimental findings
Under naïve conditions GPR55-/- deficiency is associated with bradycardia, increased diastolic LV volume and sarcomere length, cardiomyocyte size, heart weight and an immune response resembling the stretch-induced inflammation. The latter is reflected by elevated neutrophil and macrophage counts and enhanced chemokine and *Mmp-9* expression in the heart. After MI, GPR55 deficiency initially attenuates, yet subsequently prolongs acute burst in LV chemokine expression and increases cardiac abundance of pro-reparative CD206+ and MERTK+ macrophages. This in turn is accompanied by potentiated up-regulation of ECM factors such as collagen and MMP-9. Simultaneously, absence of GPR55 mitigates re-expression of the fetal gene programme and exacerbates LV remodelling culminating in an amplified functional decline post MI. Taken together, our study alludes for the first time in vivo toward a contributory role of GPR55 to development of LV volume-overload, synchronization of post-MI wound healing and regulation of LV remodelling.

Fig. S7

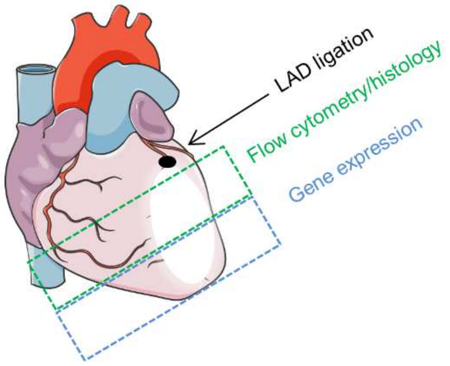

Fig. S7 Scheme of LV partition for different analyses
Myocardium below the ligation knot (and at the respective LV level in no MI and sham groups) was split for histology or flow cytometry and concurrent gene expression analyses. Herein, midventricular tissue was used for flow cytometry or histology, respectively, the apical LV was assigned to gene expression analyses.

Fig. S8

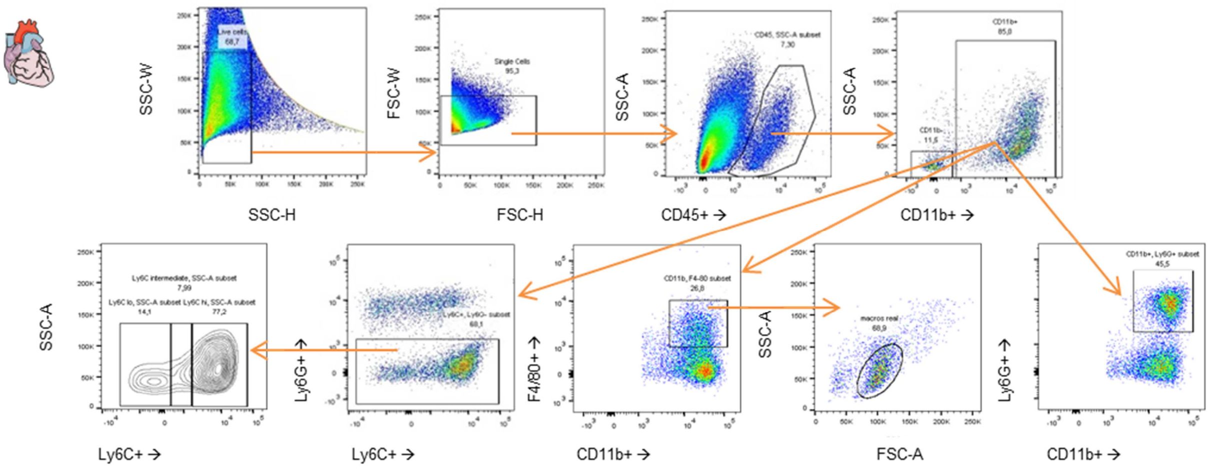

Fig. S8 Gating strategy for flow cytometric quantification of cardiac myeloid cells Leukocytes were gated as CD45+ from single cells and further sub-divided into CD11b- lymphoid and CD11b+ myeloid cells. Macrophages were identified as CD45+ CD11b+ F4/80+, monocytes as CD45+ CD11b+ Ly6G- LY6C+ and further subdivided into classical (LY6C^{high}), intermediate and non-classical monocytes (LY6C^{low}). Neutrophils were defined as CD45+ CD11b+ Ly6G+.

Supplementary Tables

Table S1 Basal phenotype of female mice aged 8 weeks, determined via echocardiography

		WT (n=8)	GPR55-/- (n=7)
IVS [mm]	diastolic	0.67 ± 0.02	0.66 ± 0.01
	systolic	0.93 ± 0.02	0.94 ± 0.03
LVAW [mm]	diastolic	0.66 ± 0.02	0.64 ± 0.02
	systolic	0.94 ± 0.03	0.94 ± 0.04
LVPW [mm]	diastolic	0.72 ± 0.02	0.70 ± 0.02
	systolic	1.07 ± 0.04	1.06 ± 0.04
LVID [mm]	diastolic	3.53 ± 0.06	3.87 ± 0.08*
	systolic	2.34 ± 0.09	2.47 ± 0.04
LVvol [µl]	diastolic	52.11 ± 2.04	64.86 ± 3.07*
	systolic	19.37 ± 0.94	21.82 ± 0.95
EF [%]		63.38 ± 2.37	66.14 ± 1.38
FS [%]		33.84 ± 1.75	35.96 ± 1.07
SV [µl]		32.74 ± 0.94	43.04 ± 2.64*
CO [ml min^{-1}]		16.88 ± 0.53	15.72 ± 0.92
Heart rate [bpm]		515 ± 5	367 ± 13*

Mean±SE; *p<0.05, student's t-test.

Table S2 Phenotype of female mice 28d post-MI, based on gravimetry

	Sham		MI	
	WT	GPR55-/-	WT	GPR55-/-
Body dimensions				
n	7	10	9	8
Body weight [g]	21.5 ± 0.4	24.5 ± 1.1#	22.6 ± 1.1	24.0 ± 0.7
Tibia length [mm]	173.3 ± 1.5	176.9 ± 2.3	169.3 ± 4.0	177.5 ± 1.4
Heart chamber weights				
n	7	10	9	8
Heart [mg]	96.9 ± 1.9	123.2 ± 9.3#	115.3 ± 9.0	128.4 ± 5.6
Atria [mg]	7.1 ± 0.5	8.6 ± 0.9	9.1 ± 1.2	9.4 ± 0.8
Wet organ weights				
n	7	10	9	8
Lungs [mg]	141.0 ± 15.0	172.9 ± 14.4	150.7 ± 14.4	145.5 ± 10.0
Liver [mg]	996 ± 35	1054 ± 45	1019 ± 38	1031 ± 97
Kidney [mg]	107.6 ± 2.5	129.4 ± 9.0#	121.3 ± 8.5	129.5 ± 5.5
Spleen [mg]	70.3 ± 3.9	102.7 ± 14.0	80.0 ± 6.4	81.4 ± 5.3
Dry organ weights				
n	7	10	9	8
Lungs [mg]	29.3 ± 0.8	32.5 ± 1.6	31.7 ± 1.3	32.4 ± 1.3
Liver [mg]	321 ± 13	340 ± 14	331 ± 14	326 ± 35
Kidney [mg]	27.7 ± 1.0	33.7 ± 2.0	30.1 ± 2.3	32.5 ± 1.3
Wet organ weights normalized to body weight [mg/g]				
n	7	10	9	8
Heart [mg]	4.5 ± 0.1	5.0 ± 0.3	5.1 ± 0.2*	5.4 ± 0.3
Lungs [mg]	6.5 ± 0.6	7.1 ± 0.5	6.7 ± 0.7	6.1 ± 0.4
Spleen [mg]	3.3 ± 0.2	4.1 ± 0.5	3.5 ± 0.3	3.4 ± 0.3
Wet organ weights normalized to tibia length [mg/mm]				
n	7	10	9	7
Heart [mg]	5.6 ± 0.1	7.0 ± 0.6	6.9 ± 0.7	7.0 ± 0.3

Lungs [mg]	0.8 ± 0.1	1.0 ± 0.1	0.9 ± 0.1	0.8 ± 0.0
Spleen [mg]	0.4 ± 0.0	0.6 ± 0.1	0.5 ± 0.1	0.5 ± 0.0

Mean±SE; $^{\#}p<0.05$ vs. corresponding WT; $^{*}p<0.05$ vs. corresponding sham, two-way ANOVA with Sidak's post hoc test.

Supplementary Methods

Tissue sampling and processing

At each study endpoint, mice were euthanized by overdosed xylazine/ketamine (120/10 mg/kg body weight) anaesthesia. EDTA blood was collected from the right ventricle and split for flow cytometry (50 µl) and plasma collection. After LV puncture and perfusion with PBS, the heart, lungs, spleen, liver, left kidney, femoral bone marrow and left tibia were excised. Wet and dry weight of lungs, liver and the left kidney were determined to assess tissue congestion. Tibia length was measured to serve as intervention independent normalization factor for heart weight. The myocardium was either entirely used for determination of infarct size or split for gene expression and histological analyses or for gene expression and flow cytometry analyses, respectively, depending on the post-MI time-point and read-outs (**see Supplementary Fig. S7**). Spleens were split longitudinally for mRNA and flow cytometry analyses. Bone marrow from one femur was used for flow cytometry. Tissue slices for histological analyses were fixed in 4% paraformaldehyde within one minute after excision of the heart and stored at 4°C. Tissue for qPCR was immediately frozen in liquid N_2 and stored at -80°C. Tissue for flow cytometric analyses was directly transferred into PBS and stored on ice until further processing.

Flow cytometry antibodies and gating strategies

All obtained cell suspensions were rinsed, re-suspended in PBS with 1% BSA, subjected to CD16/CD32 Fc-block and incubated with antibodies against murine CD11b (clone M1/70, BioLegend), CD45 (clone 30F11, BD or 104, eBioscience), CD206 (clone C068C2, BioLegend), F4/80 (clone BM8, BioLegend), Ly6C (clone HK1.4, BD), Ly6G (clone 1A8, BioLegend) and MERTK (unconjugated AF591 R&D; secondary antibody donkey anti-goat AF488, 705-545-147, Dianova). Neutrophils were identified as CD45+ CD11b+ LY6C- Ly6G+; classical monocytes as CD45+ CD11b+ CD115+ Ly6G- F4/80- LY6C^{high}; non-classical monocytes as CD45+ CD11b+ CD115+ Ly6G- F4/80- LY6C^{low}, or as CD45+ CD11b+ Ly6G- F4/80- LY6C$^{high/low}$ in the heart (**see Supplementary Fig. S8**); macrophages were gated as CD45+ CD11b+ F4/80+ and further subdivided as CD206+ or MERTK+ [43].

Data were acquired on FACS Canto II (BD Biosciences), and analyses were performed using the FloJo software (Ashland, USA).

qPCR primers

The following TaqMan probes from Applied Biosystems were used: *Il-1b* Mm00434228_m1; *Il-6* Mm00446190_m1; *Tnfα* Mm00443258_m1; *Mertk* Mm00434920_m1; *Col Iα2*; Mm00483888_m1; *Col IIIα1* Mm01254476_m1; *Nppb* Mm01255770_g1; *Nppa* Mm01255747_g1; *Myh6* Mm00440359_m1; *Myh7* Mm00600555_m1; *Gpr55* Mm02621622_s1; *Gapdh* Mm99999915_g1. Additionally, we used the following qPCR primer pairs and probes from Eurofins Genomics: *Ccl2* Fw GAGCATCCACGTGTTG, Rev TGGTGAATGAGTAGCAGCAGGT, probe AGCCAGATGCAGTTAACGCCCCACT; *Hprt* Fw GACCGGTCCCGTCATGC, Rev TCATAACCTGGTTCATCATCGC, probe ACCCGCAGTCCCAGCGTCGTG; *Il-10* Fw TTTGAATTCCCTGGGTGAGAA, Rev ACAGGGGAGAAATCGATGACA, probe TGAAGACCCTCAGGATGCGGCTG; *Mmp9* Fw CGACGACGACGAGTTGTG, Rev CATGGGGCACCATTTGAGTT, probe AAGGCGTCGTGATCCCCACTTACT.

Cardiac cell sorting

Heart cell suspensions were obtained and stained as described for flow cytometry except for using only PBS for washing and antibody dilutions. Cell suspensions were incubated with Zombie Green (BioLegend) and antibodies against murine CD45 (clone 104, BD), CD64 (X54-5/7.1, Biolegend), CD31 (clone 390, BioLegend) and CD140a (clone APA5, BioLegend). A FACSAria III (BD Biosciences) was used for sorting live (Zombie-) cardiac cells subdivided into CD45+CD64+ macrophages, CD45-CD31+CD140a- endothelial cells, CD45-CD31-CD140a+ fibroblasts and CD45-CD31-CD140a- cardiomyocytes with the 100 micron nozzle at 4-way purity precision.

Plasma albumin and volume assessment

Plasma albumin levels were determined via the Abcam Mouse Albumin ELISA kit ab207620. Plasma volume was quantified after centrifugation of 400 µl whole EDTA blood at 7500 rpm for 10 min and calculated as plasma volume per ml blood.
